# QCT-based spatio-temporal aging atlas of the proximal femur BMD and cortical geometry

**DOI:** 10.1016/j.bonr.2024.101786

**Published:** 2024-07-02

**Authors:** Alice Dudle, Yvan Gugler, Osman Berk Satir, Jan Gewiess, Stefan Klein, Philippe Zysset

**Affiliations:** a ARTORG Center for Biomedical Engineering Research, University of Bern, Switzerland; b Department of Orthopaedic Surgery and Traumatology, Inselspital, Bern University Hospital, Switzerland; c Biomedical Imaging Group Rotterdam, Department of Radiology and Nuclear Medicine, Erasmus MC University Medical Center, Rotterdam, the Netherlands

**Keywords:** Aging, Bone mineral density, Cortical thickness, CT imaging, Hip fractures, Osteoporosis

## Abstract

Aging is associated with an increased risk of fragility fractures at the hip, resulting from a loss of bone mass. While this loss is typically reported as a decreased mean areal bone mineral density (aBMD) in the proximal femur or the femoral neck, its evolution is spatially inhomogeneous, which might also contribute to the increased risk of fractures. Yet, little is known about the evolution of BMD distribution and cortical thickness with age in the proximal femur. We propose a 3D spatio-temporal atlas of the proximal femur to identify regions with high BMD losses and cortical thinning. The atlas is based on 532 post-mortem QCT scans from donors aged 20 to 94, including 179 female subjects. A point cloud with anatomically corresponding positions was defined for each femur based on a personalized coordinate system. The evolution of BMD and cortical thickness was computed as a multiple linear regression with age and BMI, for female and male subjects separately. The average BMD decrease with age was significant in all subregions for both sexes but higher in females. High BMD losses were observed in the superior and middle neck regions, in the medial part of the head, and in the trochanteric trabecular bone. BMD was well preserved in the inferior neck and, for males, in cortical regions. In both sexes, the cortical thickness decreased significantly in the superior and posterior neck cortex and increased significantly in the inferior neck. Higher BMI was associated with increased BMD in the inferior neck and medial shaft cortex, as well as with increased cortical thickness in all neck and shaft regions for both sexes. The spatio-temporal atlas showed the evolution of BMD distribution and cortical thickness in the proximal femur, with high losses in typical fracture locations, such as the femoral neck and pertrochanteric regions.

## Highlights

• 3D spatio-temporal atlas of the aging femur based on QCT scans
• Decreased BMD and cortical thickness with age in the superior femoral neck
• Stable BMD and increased cortical thickness with age in the inferior femoral neck
• Increased BMD and cortical thickness with BMI in most cortical regions

## Graphical abstract

**Figure:**
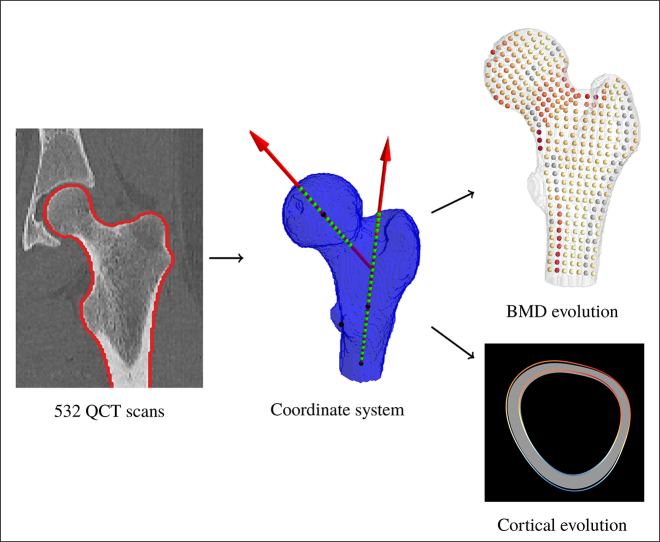

## 1 Introduction

Bone mineral density (BMD) at the human proximal femur evolves with age. In the current clinical practice, dual-energy x-ray absorptiometry (DXA) is used to quantify areal bone mineral density (aBMD) at the hip and spine and diagnose osteoporosis. Low bone density, and osteoporosis in particular, are associated with an increased risk of fragility fractures. Such fractures, particularly at the hip, are associated with an increased mortality risk, a loss in quality of life, and high medical costs. Hip fractures affect more than one in five women and around one in fourteen men. They affect the patient's quality of life and increase mortality risk (Kanis et al., 2021; Guzon-Illescas et al., 2019).

The evolution of aBMD with age has been studied extensively and characterized by reference datasets (Looker et al., 1998). Females and males reach a peak bone mass at the femoral neck around 19 and 20 years, respectively (Xue et al., 2020; Berger et al., 2010). This peak is followed by a slow decrease (0.3–0.5 % loss per year), which becomes stronger around menopause for females (0.6–0.7 % between 45 and 75 years). Interestingly, the bone loss rate is higher in the femoral neck than in the total proximal femur, showing that the density distribution also evolves with age (Looker et al., 1998). Indeed, load-bearing regions tend to preserve bone longer during aging, while regions that experience little mechanical loading lose bone faster (Christen et al., 2014). Therefore, the bone becomes more likely to fracture if loaded in an unusual direction, for example, during a fall. The inhomogeneous density evolution is thus relevant for the prediction of fragility fractures. However, DXA-based T-scores do not account for the detailed spatial distribution of density in the bone, which restricts the fracture risk estimation. Moreover, body mass index (BMI) correlates positively with BMD, and low BMI patients have a substantially higher fracture risk (De Laet et al., 2005). Therefore, we can hypothesize that high BMI subjects are protected against femoral fractures by an increased BMD, at least in critical fracture regions. However, few studies have investigated the relationship between BMI and the spatial distribution of BMD in the proximal femur.

The evolution of aBMD distribution has been investigated by Farzi et al. (2020) based on 2D DXA scans from over 13,000 female subjects. They found that higher BMI was associated with increased aBMD at the diaphysis and in Ward's triangle, which is a subregion of the neck. Their atlas also showed aBMD loss with age in all regions, in particular, a reduction of the shaft cortical thickness and a substantial aBMD decrease in the trochanteric and femoral neck regions. One exception was the inferior neck cortex, which was well preserved with age. However, DXA scans are 2D projections of the BMD distribution. Therefore, every aBMD measurement is the sum of all densities along the projection line. Since most aBMD values contain a mixture of cortical and trabecular bone, density variations are challenging to interpret. While the 2D atlas gives some insights into the aging process in the proximal femur, including the third dimension is crucial to understanding the evolution of the spatial BMD distribution and cortical thickness. In addition, the full 3D BMD distribution in the proximal femur is also required to conduct realistic finite element analyses and derive the bone's mechanical properties.

Other studies were based on 3D QCT scans of the femur but focussed only on the femoral neck (Johannesdottir et al., 2013; Khoo et al., 2019; Poole et al., 2010; Mayhew et al., 2005). They found that BMD and cortical thickness decreased faster in the superior than in the inferior part of the neck for both males and females and that the loss was higher in females than males. While most studies reported that the inferior neck cortex was preserved with age, one study even found an increase of thickness in this region, maybe thanks to more detailed measurements (Mayhew et al., 2005). In addition, the inferior cortical thickness was associated with body weight (Johannesdottir et al., 2013). Another study focused on the cortical thickness in the diaphysis and found larger thicknesses in male than female and in younger than elderly subjects, except for the medial region (Someya et al., 2020). Finally, one team considered the whole proximal femur and compared the 3D BMD distribution between three groups of female subjects at different ages (Carballido-Gamio et al., 2013). Substantial differences between age groups were identified in the superior region of the neck, the medial part of the head, and the trabecular bone of the trochanteric region. Conversely, BMD was well preserved in the inferior part of the neck, the superolateral part of the head, the medial and lateral regions of the diaphysis, and the upper greater trochanteric region. However, this study did not consider the BMD variations associated with BMI or in males.

The present study aims to develop a 3D spatio-temporal atlas of BMD distribution as a function of age and BMI for both males and females. In addition, we investigate the evolution of the cortical thickness in the femoral neck and shaft. To build a robust pipeline applicable to any femur geometry, we took advantage of the personalized coordinate system developed in a previous work (Dudle et al., 2023). This allowed us to obtain anatomical correspondence between the femurs without relying on image registration. Therefore, the risk of high local deformations and BMD interpolation errors due to non-rigid image registration was avoided. In addition, the personalized coordinate system enabled us to retrieve BMD values and geometrical information directly from the original QCT images. The cortical thickness was extracted with a pipeline developed and validated by Treece and Gee (2015) and freely available in the software Stradview.^1^

In comparison to previous studies, this work also stands out by the number of included images (532 CT scans), which were scanned on a single CT device with a calibration phantom. In addition, the dataset covers the whole adult age range (20–94) for both female and male subjects.

## 2 Materials and methods

### 2.1 Dataset

We used a dataset of 816 whole-body post-mortem CT scans, collected between 2015 and 2021 at the Forensic Institute of the University of Bern, as part of the autopsy procedure. The scans were performed on a SOMATOM Definition AS 64 CT scanner (Siemens, Munich, Germany), with a calibration phantom (QRM-BDC6 from QRM GmbH, Mohrendorf, Germany) included in the scan table. Important scanning parameters, such as the voltage (140 kV), the slice thickness (0.7 mm), and the reconstruction kernel (I70f\3), were constant for all scans. The in-plane voxel size ranged from 0.80 to 1.52 mm. Scans were reviewed by an orthopedist to exclude all cases with relevant image artifacts, aberrant bone morphology (deformations, lesions, fractures), or surgical implants. Additional exclusion criteria were young age (<20 years), unknown age, severely underweight (BMI < 16) or severely obese (BMI > 40) donors. After exclusion, 532 donors were included in the study, covering wide age and BMI ranges, as detailed in Table 1 and in Fig. 9 in the appendix. Left femurs were mirrored along the sagittal plane to match the geometry of right femurs. Both femurs of each donor were included in the analysis, resulting in 1064 cases. The study was approved by the local ethical committee.

**Table 1:** Dataset description. Included cases with age, body mass index (BMI), and average bone mineral density (BMD) in the proximal femur.

	N	Age			BMI [kg/m^2^]			BMD [mg/cm^3^]		
		Avg	Min	Max	Avg	Min	Max	Avg	Min	Max
Female	179	56	21	94	25	17	40	375	143	546
Male	353	53	20	94	26	17	40	390	209	569

### 2.2 Image preparation

The voxel values were converted from Hounsfield units to BMD using the calibration phantom as a reference. In short, the six chambers of the phantom correspond to different concentrations of hydroxyapatite ranging from 0 to 800 mg/cm^3^. The chambers were detected automatically with a circle detection algorithm, and voxel values were averaged in a circular region covering 80 % of the detected radius to avoid partial volume effects. A calibration equation was then computed by fitting a linear curve between Hounsfield and BMD values. The calibration was performed separately for each slice to account for current modulation.

**Fig. 1:**
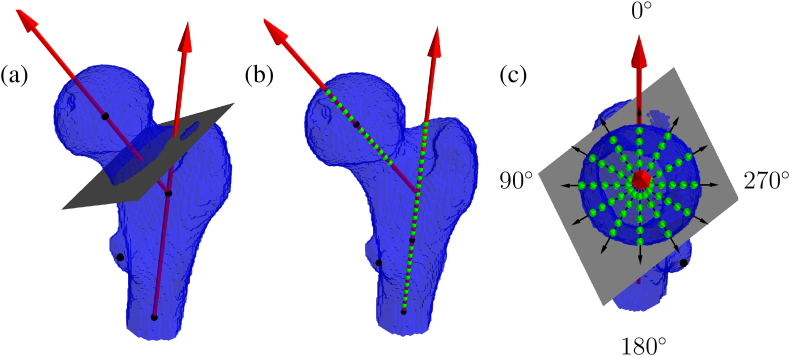
Personalized coordinate system and creation of the point cloud. (a) Neck and shaft axes with cutting plane separating regions. (b) Discretization of both axes in a finite number of points. (c) Definition of 12 directions and discretization in finite numbers of points.

An automated segmentation of the osseous pelvis and bilateral femoral regions was developed. Segmenting both regions simultaneously produced better results than segmenting the femoral regions alone, especially in the acetabulum region. Initially, the proximal femurs and the pelvis were segmented manually in 18 scans using two different labels. These images were then used to train a neural network based on the nnU-net method (Isensee et al., 2021). All images were then segmented using the trained model, and the results were inspected visually. Whenever corrections were deemed necessary, the femur segmentation was adapted manually (<10% of cases). Based on the segmentation results, a surface mesh was obtained for each femur with the marching cube algorithm (see Fig. 1).

### 2.3 Personalized coordinate system and point cloud

A coordinate system was derived for each femur in a personalized way, following the method described in a previous work (Dudle et al., 2023). In short, two intersecting axes were defined, one passing through the femoral neck and one through the shaft, as illustrated in Fig. 1 (a). In addition, the center of the femoral head and the tip of the lesser trochanter were detected. The proximal femurs were cropped at a distal level positioned with respect to the lesser trochanter, using the femur volume above the lesser trochanter as a scaling measure.

Using the coordinate system, we defined a point cloud in cylindrical coordinates $\left(r,\phi ,z\right)$, anatomically corresponding between all proximal femurs. First, the proximal femur was divided into two regions by a cutting plane orthogonal to the neck axis. We computed the cross-section area at different positions along the neck axis and positioned the plane in the most lateral cross-section before the strong area increase due to the greater trochanter (see Fig. 1 (a)). The point cloud was described by the neck axis on the proximal side of the cutting plane and by the shaft axis on the distal side.

Both axes were discretized into a fixed number of points, as illustrated in Fig. 1 (b). Each discrete point corresponded to a z value in cylindrical coordinates and defined a plane orthogonal to the corresponding axis, with z=0 at the intersection between the head surface and the neck axis, z=12 at the intersection between the cutting plane and the neck axis, z=13 and z=38 at the most superior and distal positions along the shaft axis, respectively. The head center (z=5), the intersection between the cutting plane and the superior neck surface (z=15), and the level of the lesser trochanter (z=28) were used as intermediate reference points to account for different neck lengths, greater trochanter shapes, and lesser trochanter positions. Remaining positions were placed equidistantly between fixed points.

The orthogonal planes were divided in 12 directions or angles ϕ, in 30° steps counterclockwise (see Fig. 1 (c)). Along the neck axis, the zero angle direction ($\phi =0^{{}^{\circ}}$) was defined as the shaft axis projection onto the orthogonal planes, pointing in the superior direction. Along the shaft axis, the zero angle direction was defined based on the neck axis projection onto the orthogonal planes so that $\phi =0^{{}^{\circ}}$ pointed in the lateral direction and $\phi =180^{{}^{\circ}}$ in the medial direction. In the lesser trochanter region, the 8th angular direction was constrained to pass through the lesser trochanter landmark (instead of $\phi =210^{{}^{\circ}}$). The four remaining directions between the 8th and $\phi =0^{{}^{\circ}}$ directions were distributed at equal angles.

Equidistant points were then placed between the axis (r=0) and the surface of the femur (r=1), as shown in Fig. 1 (c). The number of points was set for each pair $\left(\phi ,z\right)$ to get a uniform spacing for an average femur geometry. In the intertrochanteric region, the cutting plane replaced the surface of the femur when appropriate. In the greater trochanter, some directions (ϕ,z) had more than one intersection with the femur surface. In that case, the intersection furthest from the axis was chosen as r=1. Points were distributed equidistantly in between, even if some points were outside the femur. Outside points were discarded in the analysis, as described below.

### 2.4 BMD analysis

For each point of the cloud, the BMD was averaged within a spherical region to reduce the noise. The sphere radius was proportional to the femur size so that the coverage of the femur volume by the point cloud was independent of the femur size. The sphere radius measured 1.5 mm on average (range 1.2–1.8 mm), which was a compromise between noise reduction and detailed spatial resolution. If the sphere intersected with the femur surface, only the sphere section within the femur was used in the average. Spheres placed outside the femur or with less than five voxels within the femur were dismissed. If a sphere was dismissed in >20 % of the cases, the point was removed from the atlas.

**Fig. 2:**
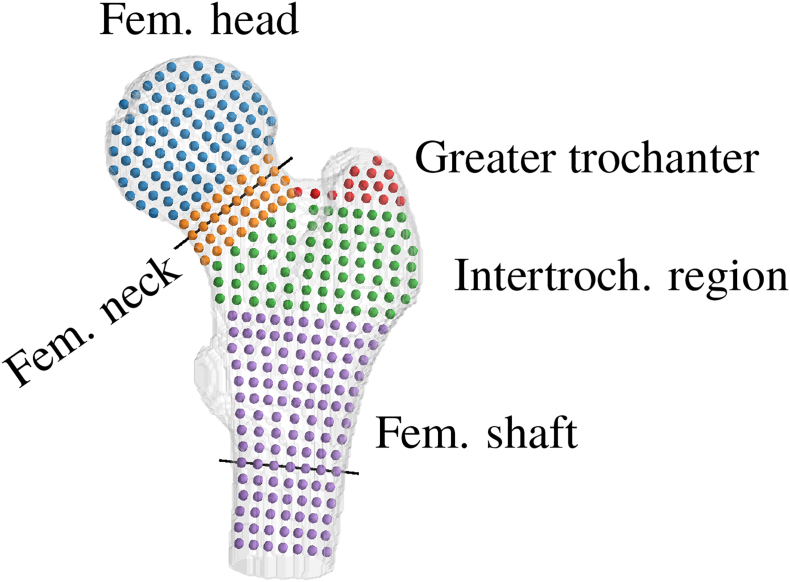
Point cloud. Slice $\phi \in \left\{0^{{}^{\circ}},180^{{}^{\circ}}\right\}$ and ROIs for the BMD analysis. The evolution of the cortical geometry will be illustrated for the two locations marked by black lines.

For each point $\left(r,\phi ,z\right)$ in the cloud, a multiple linear regression was fitted for females and males separately:

(1) $$\begin{array}{l} {\mathrm{BMD}}_{\left(r,\phi ,z\right)}^{w}=\mathrm{age}\times q_{\left(r,\phi ,z\right)}^{w}+\mathrm{BMI}\times s_{\left(r,\phi ,z\right)}^{w}+b_{\left(r,\phi ,z\right)}^{w}\\ {\mathrm{BMD}}_{\left(r,\phi ,z\right)}^{m}=\mathrm{age}\times q_{\left(r,\phi ,z\right)}^{m}+\mathrm{BMI}\times s_{\left(r,\phi ,z\right)}^{m}+b_{\left(r,\phi ,z\right)}^{m} \end{array}$$

where q and s are the slopes for age and BMI, respectively, and b is the intercept. The relationship with age or BMI was considered significant if the corresponding *p*-value was below 0.05. These regressions were used to generate the BMD distribution in the proximal femur for different ages and BMI values for both sexes. We also computed the average evolution for different regions of interest (ROIs) illustrated in Fig. 2 by computing an additional regression on the averaged BMD measures.

### 2.5 Evolution of the cortical thickness

The 3D coordinate system also allowed us to observe the evolution of the cortex geometry. The endosteal cortical surface was segmented using the cortical bone mapping algorithm (CBM v2) of the Stradview software^2^ (University of Cambridge) (Treece and Gee, 2015). As input, we used the soft kernel reconstruction (I31f\3) of the same scans and the mesh of the outer cortical surface generated previously. However, the resulting endosteal surface was not satisfactory in the femoral head region, probably due to the subvoxel thickness of the cortical layer. Therefore, our analysis focused on the femoral neck and shaft regions. In these two regions, we searched for the intersection with both the inner and the outer cortical meshes along each direction $\left(\phi ,z\right)$ of the coordinate system. The inner and outer radii were defined as the distance between the axis and the respective intersection, and the cortical thickness as the difference between both radii.

For each direction $\left(\phi ,z\right)$ in the neck and shaft regions, we computed multiple linear regressions with age and BMI for the inner radius, the outer radius, and the cortical thickness, respectively, to model the evolution of the cortex geometry. To summarize the variations in a few ROIs, the cortical thickness was averaged over the neck ($z\in \left[912\right]$) and diaphysis ($z\in \left[3338\right]$) regions in anatomical quadrants (anterior: $\phi \in \left\{60^{{}^{\circ}},90^{{}^{\circ}},120^{{}^{\circ}}\right\}$, inferior/medial: $\phi \in \left\{150^{{}^{\circ}},180^{{}^{\circ}},210^{{}^{\circ}}\right\}$, posterior: $\phi \in \left\{240^{{}^{\circ}},270^{{}^{\circ}},300^{{}^{\circ}}\right\}$ and superior/lateral: $\phi \in \left\{330^{{}^{\circ}},0^{{}^{\circ}},30^{{}^{\circ}}\right\}$). A multiple linear regression was fitted for each region to determine the quantitative evolution of the cortical thickness with age and BMI.

## 3 Results

### 3.1 BMD regression and visualization of the atlas

**Fig. 3:**
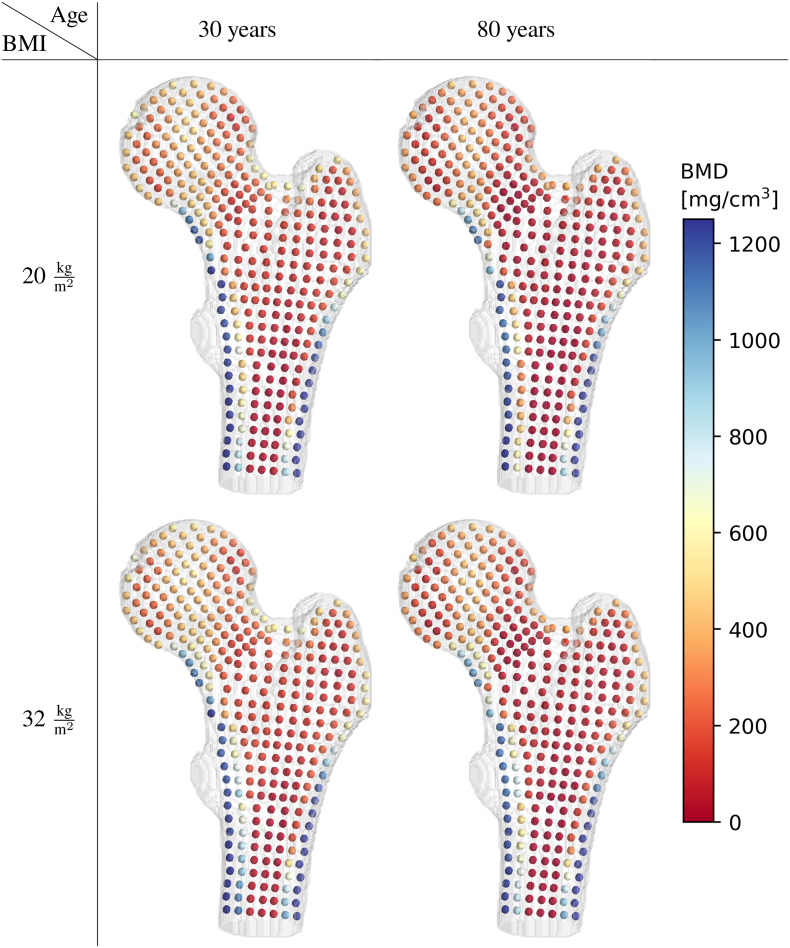
Spatio-temporal atlas of the proximal femur in female subjects. BMD distribution [mg/cm^3^] generated from the atlas for various ages and BMI values, illustrated for the cross-sectional slice $\phi \in \left\{0^{{}^{\circ}},180^{{}^{\circ}}\right\}$. The value at each position $\left(r,\phi ,z\right)$ corresponds to ${\mathrm{BMD}}_{\left(r,\phi ,z\right)}^{w}$ in eq. 1.

The point cloud consisted of 1742 anatomically corresponding positions distributed in the proximal femur. The overlap between spheres was typically between 2 and 4 % of the sphere volume, with a maximum of 5.6 %. For the BMD analysis, 1742 multiple linear regressions were computed for both females and males. For females, 1607 correlated significantly with age and 862 with BMI. For males, 1440 regressions showed a significant correlation with age and 924 with BMI. The multiple linear regression results can be used to generate the BMD distribution for any age and BMI values, as illustrated in Fig. 3 for females and in Fig. 10 in the appendix for males.

### 3.2 Evolution of the BMD distribution

**Fig. 4:**
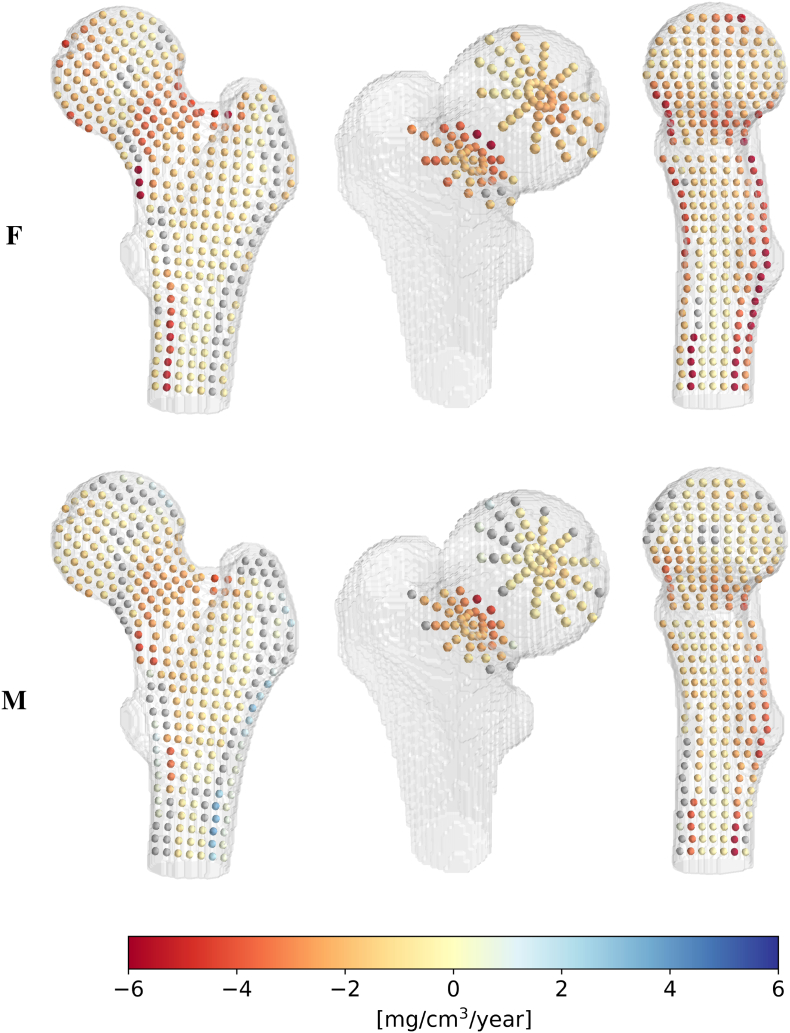
BMD evolution with age in the proximal femur. BMD difference per year [mg/cm^3^/year] in females (upper row) and males (lower row): $q_{\left(r,\phi ,z\right)}^{w}$ and $q_{\left(r,\phi ,z\right)}^{m}$ from eq. 1. Gray spheres represent non-significant correlations between age and BMD $\left(p>0.05\right)$. From left to right, the columns correspond to cross-sections $\phi \in \left\{0^{{}^{\circ}},180^{{}^{\circ}}\right\}$, $z\in \left\{312\right\}$ (medial head and mid-neck) and $\phi \in \left\{90^{{}^{\circ}},270^{{}^{\circ}}\right\}$.

To identify high-loss regions, the BMD difference per year is shown in Fig. 4 for females and males. Significant BMD losses appear in the posterior, middle, and superior neck regions and are more pronounced in females than males. This is also the case in the medial part of the head, although with a slower BMD loss. A low but significant BMD loss is observed in the trabecular bone of the intertrochanteric and shaft regions in both sexes. In addition, females suffer from BMD losses in the inferior neck cortex and the endocortical layer of the shaft, except on the lateral side. In males, the shaft and inferior neck cortex are well preserved, as shown by the many positions with non-significant correlations or slightly positive slopes. Interestingly, we also observe a vertical line of non-significant correlations through the head in males.

**Fig. 5:**
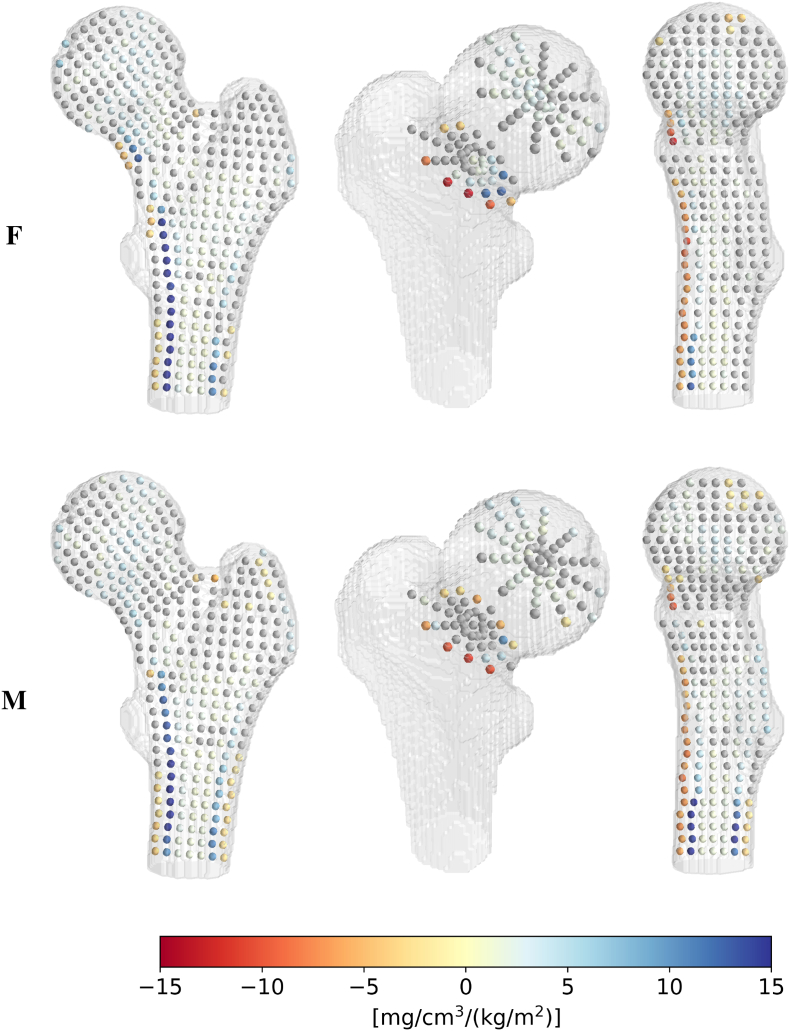
BMD evolution with BMI in the proximal femur. BMD difference per BMI point [(mg/cm^3^)/(kg/m^2^)] in females (upper row) and males (lower row): $s_{\left(r,\phi ,z\right)}^{w}$ and $s_{\left(r,\phi ,z\right)}^{m}$ from eq. 1. Gray spheres represent non-significant correlations between BMI and BMD $\left(p>0.05\right)$. From left to right, the columns correspond to cross-sections $\phi \in \left\{0^{{}^{\circ}},180^{{}^{\circ}}\right\}$, $z\in \left\{312\right\}$ (medial head and mid-neck) and $\phi \in \left\{90^{{}^{\circ}},270^{{}^{\circ}}\right\}$.

Similarly, Fig. 5 illustrates the BMD difference per BMI point. For both females and males, the highest BMD increases in are observed in the endocortical layer of the shaft, in particular on the medial side. In females, a similar effect also appears in the inferior neck cortex.

### 3.3 BMD in regions of interest

**Fig. 6:**
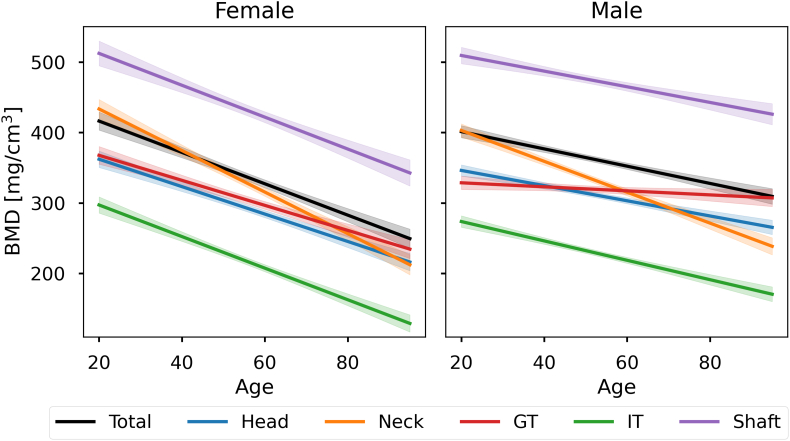
Average BMD evolution in various ROIs. Linear regression between age and BMD for the total proximal femur, the femoral head, neck, greater trochanter (GT), intertrochanteric region (IT), and shaft.

The predicted evolution of BMD with age is shown in Fig. 6 for a fixed BMI value (25 kg/m^2^). The highest losses with age are observed in the femoral neck (−2.9 and 2.2 mg/cm^3^/year) and intertrochanteric region (−2.2 and 1.4 mg/cm^3^/year) for females and males. The relative BMD differences are quantified in Table 2. In both sexes, we observe a significant variation with both age and BMI in the total proximal femur. All subregions show greater differences with aging in females than in males. The femoral shaft shows the largest increase with BMI in both sexes, followed by the femoral head. Other subregions are not significantly associated with BMI.

**Table 2:** Quantitative BMD evolution for different ROIs. Relative BMD differences per year and BMI point with respect to the predicted BMD for an age of 20 and a BMI of 25. (${}^{*}p<0.05$, ${}^{**}p<0.01$).

	Age		BMI	
	Female	Male	Female	Male
Total prox. femur	$-0.53{\%}^{**}$	$-0.31{\%}^{**}$	$+0.33{\%}^{**}$	$+0.36{\%}^{**}$
Femoral head	$-0.54{\%}^{**}$	$-0.31{\%}^{**}$	$+0.35{\%}^{**}$	$+0.35{\%}^{**}$
Femoral neck	$-0.68{\%}^{**}$	$-0.54{\%}^{**}$	+0.14%	−0.04%
Greater trochanter	$-0.48{\%}^{**}$	$-0.09{\%}^{*}$	−0.09%	−0.08%
Intertroch. region	$-0.75{\%}^{**}$	$-0.50{\%}^{**}$	+0.24%	+0.13%
Femoral shaft	$-0.44{\%}^{**}$	$-0.22{\%}^{**}$	$+0.41{\%}^{**}$	$+0.53{\%}^{**}$

### 3.4 Evolution of the cortical thickness

**Fig. 7:**
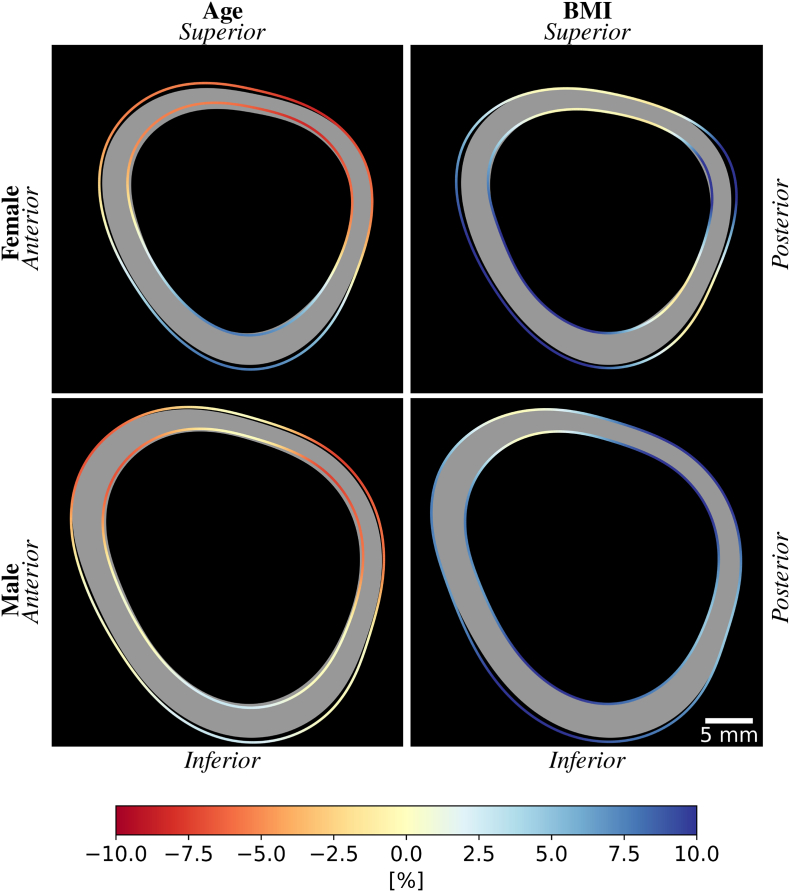
Evolution of the cortex in the femoral neck with age and BMI for females (top) and males (bottom). The gray area represents the predicted shape of the neck cortex at age 20 and a BMI of 25. The colored lines define the inner and outer cortical surfaces for an increase of 20 years (left) or 10 points of BMI (right). The colors illustrate the relative difference in cortical thickness [%].

The evolution of the cortical geometry in the femoral neck is illustrated in Fig. 7. The average evolution was estimated along 12 directions and interpolated with a B-spline curve for better visualization. In females, the inner and outer radii grow larger with age and BMI in almost all regions. In the superior neck, the inner radius increases faster with age than the outer one, resulting in a thinning of the cortex. The opposite happens in the inferior neck, where the cortex becomes thicker with age. The cortical thickness also increases for females with larger BMI values, especially in the superior-posterior and inferior-anterior directions.

The inner and outer radii show less variation with age or BMI in males than in females. The age-related differences are concentrated in the superior-posterior part, where the cortex becomes thinner, and in the inferior part, where both radii increase, but the thickness remains stable. Males with an increased BMI value show a thicker cortex in all directions except the superior-anterior part.

**Fig. 8:**
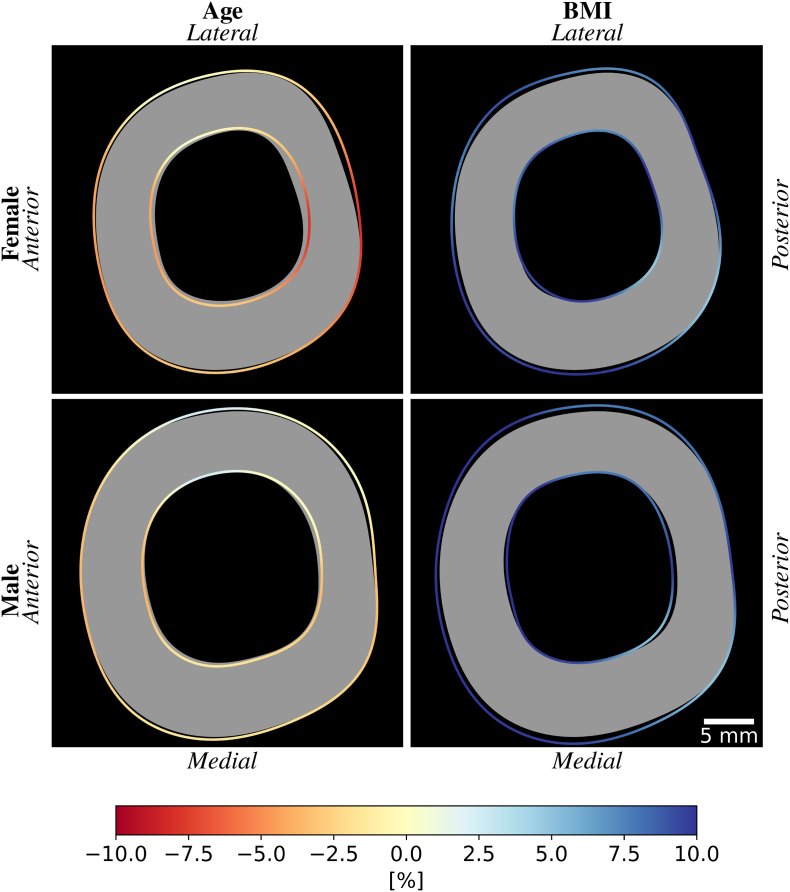
Evolution of the cortex in the femoral shaft with age and BMI for females (top) and males (bottom). The gray area represents the predicted shape of the neck cortex at age 20 and a BMI of 25. The colored lines define the inner and outer cortical surfaces for an increase of 20 years (left) or 10 points of BMI (right). The colors illustrate the relative difference in cortical thickness [%].

Corresponding results for the femoral shaft cortex are shown in Fig. 8. In females, the inner and outer radii increase with age in almost all directions, but the thickness decreases mainly in the posterior direction. Males show little variations in the shaft cortical geometry with age. Increased BMI in females and males results in a larger outer radius, smaller inner radius, and thus a thicker cortex in all directions.

The relative thickness differences are quantified in Table 3 for the neck and shaft cortex. The differences with age are larger for females than males in all regions except the anterior neck. The thickness decreases significantly with age in most regions, except for a significant increase in the lower neck and non-significant variations in the lateral shaft and female anterior neck. The thickness increases significantly with BMI in all regions for females and males, except for the female upper neck. The largest increases are found in the female anterior neck. In the shaft, the increase with BMI is homogenous in all quadrants for females and males.

**Table 3:** Quantitative evolution of the cortical thickness for different ROIs. Relative cortical thickness differences per year and BMI point with respect to the predicted cortical thickness at age 20 and a BMI of 25. (${}^{*}p<0.05$, ${}^{**}p<0.01$).

	Age		BMI	
	Female	Male	Female	Male
Upper neck	$-0.33{\%}^{**}$	$-0.20{\%}^{**}$	+0.34%	$+0.69{\%}^{**}$
Anterior neck	−0.04%	$-0.14{\%}^{**}$	$+1.03{\%}^{**}$	$+0.86{\%}^{**}$
Lower neck	$+0.30{\%}^{**}$	$+0.10{\%}^{*}$	$+0.94{\%}^{**}$	$+0.91{\%}^{**}$
Posterior neck	$-0.25{\%}^{**}$	$-0.24{\%}^{**}$	$+0.63{\%}^{**}$	$+0.55{\%}^{**}$
Lateral shaft	−0.07%	+0.05%	$+0.82{\%}^{**}$	$+0.84{\%}^{**}$
Anterior shaft	$-0.21{\%}^{**}$	$-0.15{\%}^{**}$	$+0.81{\%}^{**}$	$+0.97{\%}^{**}$
Medial shaft	$-0.18{\%}^{**}$	$-0.07{\%}^{**}$	$+0.87{\%}^{**}$	$+0.80{\%}^{**}$
Posterior shaft	$-0.33{\%}^{**}$	$-0.13{\%}^{**}$	$+0.77{\%}^{**}$	$+0.75{\%}^{**}$

## 4 Discussion

In this work, we developed a 3D atlas of BMD distribution and cortical thickness in the proximal femur for both females and males. Although the results at age 20 were similar in both sexes, females showed an overall higher loss of BMD and cortical thickness with age, which is coherent with the higher fracture rates in older females. The average BMD decrease with age was significant for both sexes in all subregions. The femoral neck showed a BMD loss of 0.68 % and 0.54 % per year in females and males, respectively, coherently with previous work (Keaveny et al., 2010).

In accordance with existing 2D and 3D studies in female subjects, we obtained a substantial BMD decrease in the superior and middle neck regions, while the inferior neck was better preserved with age (Farzi et al., 2020; Johannesdottir et al., 2013; Khoo et al., 2019; Carballido-Gamio et al., 2013). This reflects the strain distribution obtained by Kersh et al. (2018) in the femoral neck for daily activities such as walking, where the deformations were higher in the inferior than in the superior neck. Higher strains are believed to trigger bone formation and thus result in better bone preservation with age. We also observed BMD loss in the medial part of the head and the trabecular bone of the trochanteric region, which is coherent with the study by Carballido-Gamio et al. (2013).

We are not aware of any equivalent 3D results for the whole proximal femur BMD in males. In this study, we found that regions suffering from BMD loss were generally the same as in females but that the BMD decrease was slower. Males also showed well-preserved bone in cortical regions and in a vertical line through the head, which might correspond to the load-bearing region in the stance position.

In both sexes, the cortical thickness decreased significantly in the superior and posterior neck cortex, while a significant increase was observed in the inferior neck. While most previous studies showed higher losses in the superior than in the inferior neck cortex (Johannesdottir et al., 2013; Poole et al., 2010), we found only one study demonstrating a similar increase in the lower part (Mayhew et al., 2005). This difference could be explained by the choice of angular sections for the thickness analysis, whose definitions vary between studies. A localized thickness increase might thus be missed or averaged out depending on the position and size of the sections. In the shaft, the cortical thickness decreased significantly with age in all quadrants, except in the lateral part, with the largest decrease observed in the posterior cortex in females. This is coherent with the BMD differences observed and partly with the results by Someya et al. (2020). Indeed, they also observed the largest thickness decrease in the female posterior region, but the smallest decrease was found on the medial side for both sexes. However, the region defined as proximal diaphysis in their study is considerably larger than the one used here, which might explain the difference.

Higher BMI was associated with increased BMD in the inferior neck cortex and increased cortical thickness in all neck regions for both sexes. In females, this thickness increase was particularly pronounced in the distal and anterior neck cortex. This effect might be protective against femoral neck fractures, as patients with high BMI are known to have a lower fracture risk. Johannesdottir et al. (2013) found an association between body weight and inferior cortical thickness in the neck but did not report results for BMI. Apart from the femoral neck, the effect of BMI on the 3D BMD distribution and cortical thickness had not been investigated by previous studies, to the best of our knowledge. We found an increased BMD in the endocortical layer of the femur shaft and an increased cortical thickness in the same region in both males and females with high BMI.

Our study was limited by the lack of medical history of the donors due to the forensic context of the image collection. However, all cases presenting potential bone pathologies were assessed by an orthopedist. Although scans with visible air bubbles were excluded, we can not exclude that some cases with small, subvoxel-sized bubbles were included in the analysis. Nonetheless, the average over several voxels should reduce the impact of such cases. Moreover, this study only considered a linear model, although the BMD and cortical thickness evolution might be non-linear. This approximation was deemed appropriate after visual inspection of the regression results (see Figs. 11–14 in the appendix) and considering the dataset size. Furthermore, the dataset was unbalanced with regard to sex, with 34 % of female and 66 % of male subjects. Since sufficient data points were collected for each sex, two separate analyses could be conducted. Thus, the imbalance did not affect the results. Due to the large age range, secular variations might occur between the different generations. Unfortunately, they are indistinguishable from age-related differences in a cross-sectional study. Further analysis would require a longitudinal study.

Finally, the analysis was conducted independently of bone size. Therefore, our results do not show differences in BMD or cortical thickness related to bone size.

In future work, the atlas could be combined with finite element analysis to assess whether positions with well-preserved BMD and cortical thickness correspond to load-bearing regions in physiological conditions. Our method could also be used to evaluate osteoporosis treatments by identifying femoral regions where bone formation is triggered, or bone loss is slowed down.

## 5 Conclusion

The atlas developed in this work can generate the 3D BMD distribution and cortical geometry in the proximal femur for any adult age and BMI value for both females and males. It also highlights the regions with age-related losses and regions associated with BMI in each sex. Thereby, it improves our understanding of the increased fracture risk with aging, particularly in female subjects, and with low BMI.

## CRediT authorship contribution statement

**Alice Dudle:** Writing – original draft, Visualization, Software, Methodology, Data curation, Conceptualization. **Yvan Gugler:** Writing – review & editing, Software, Methodology. **Osman Berk Satir:** Writing – review & editing, Software, Methodology. **Jan Gewiess:** Writing – review & editing, Data curation. **Stefan Klein:** Writing – review & editing, Supervision. **Philippe Zysset:** Writing – review & editing, Supervision, Funding acquisition, Conceptualization.

## Declaration of competing interest

The authors have no competing interests to declare.

## Data Availability

Data will be made available on request.

## Appendix A Supplementary data

Supplementary figures Image 1

## Footnotes

1 https://mi.eng.cam.ac.uk/Main/StradView

2 https://mi.eng.cam.ac.uk/Main/StradView
