# Supplementary material for: QCT-based spatio-temporal aging atlas of the proximal femur BMD and cortical geometry
Source: Bone Rep. 2024 Jul 2;22:101786. doi: 10.1016/j.bonr.2024.101786 (PMC11639439; doi:10.1016/j.bonr.2024.101786)
Supplement: Supplementary file 1 — Supplementary figures [file mmc1.pdf]

Appendix

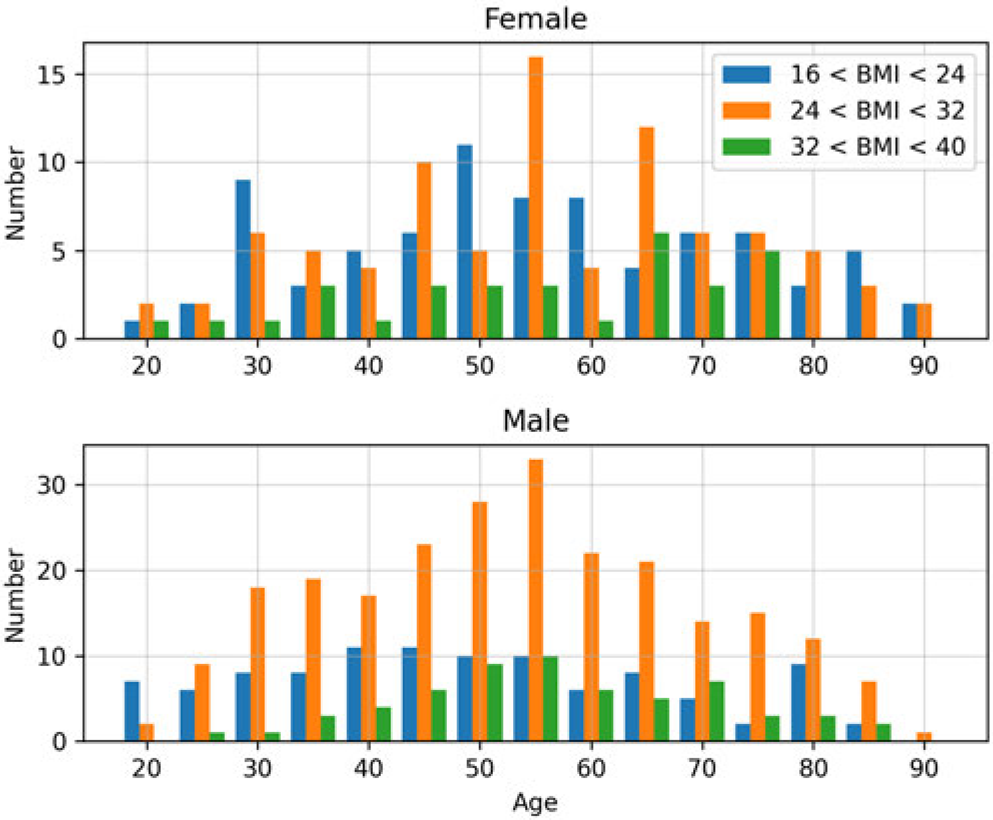

Figure 9: Distribution of age and BMI in the dataset.

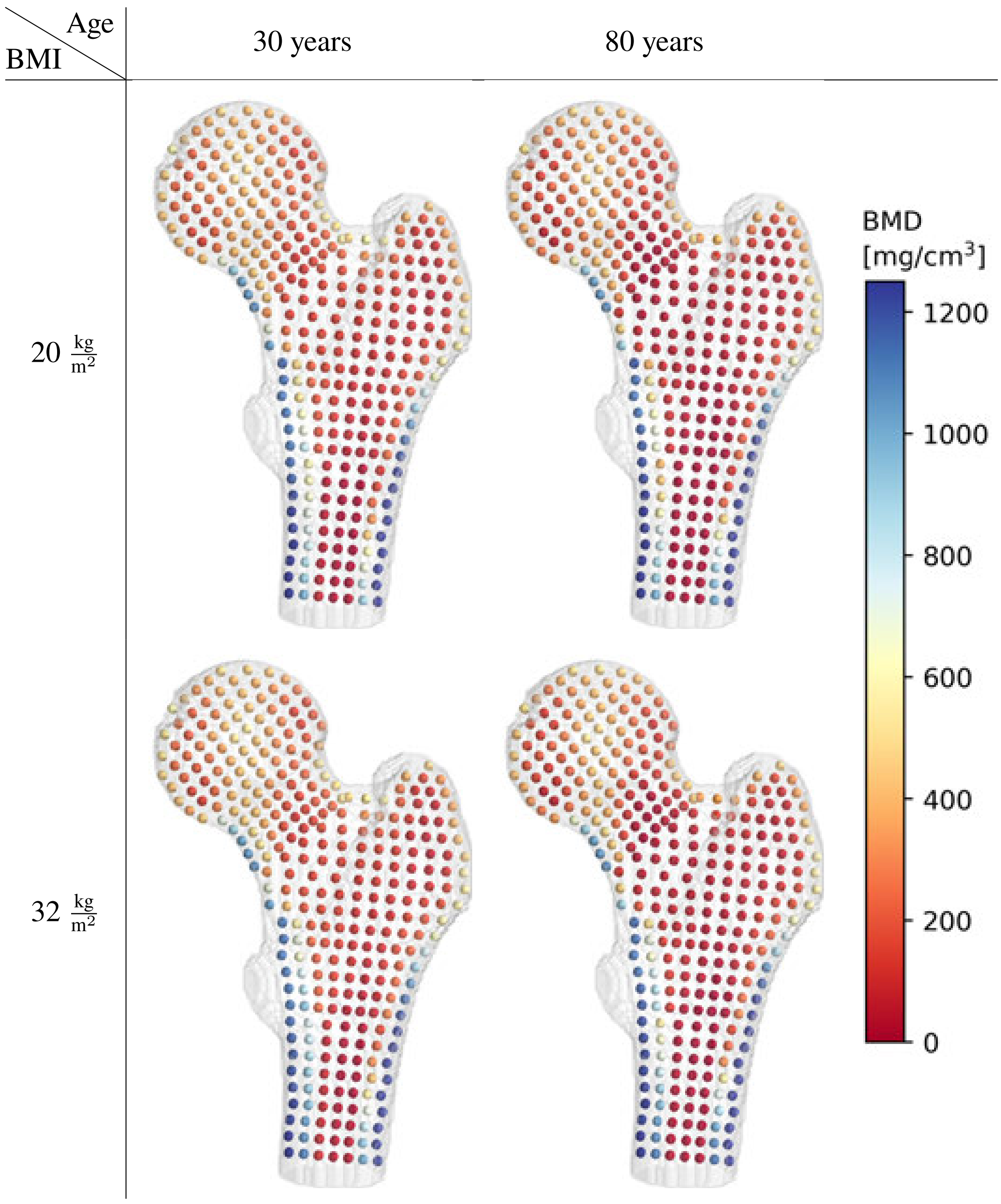

Figure 10: Spatio-temporal atlas of the proximal femur in male subjects. BMD distribution [mg/cm^3] generated from the atlas for various ages and BMI values, illustrated for the cross-sectional slice $\phi \in \{0°, 180°\}$. The value at each position (r, ϕ, z) corresponds to $\text{BMD}^m_{(r,\phi,z)}$ in equation 1.

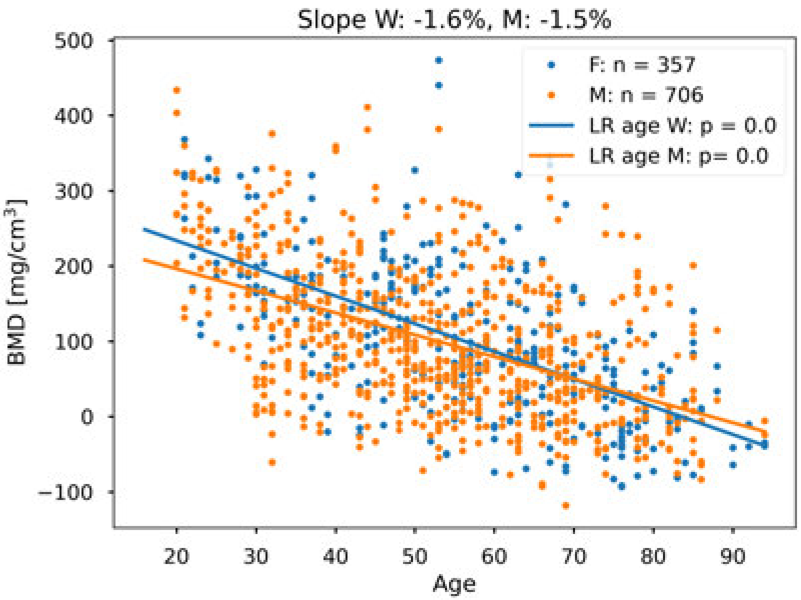

Figure 11: Linear regression between BMD and age for a point in the femoral neck.

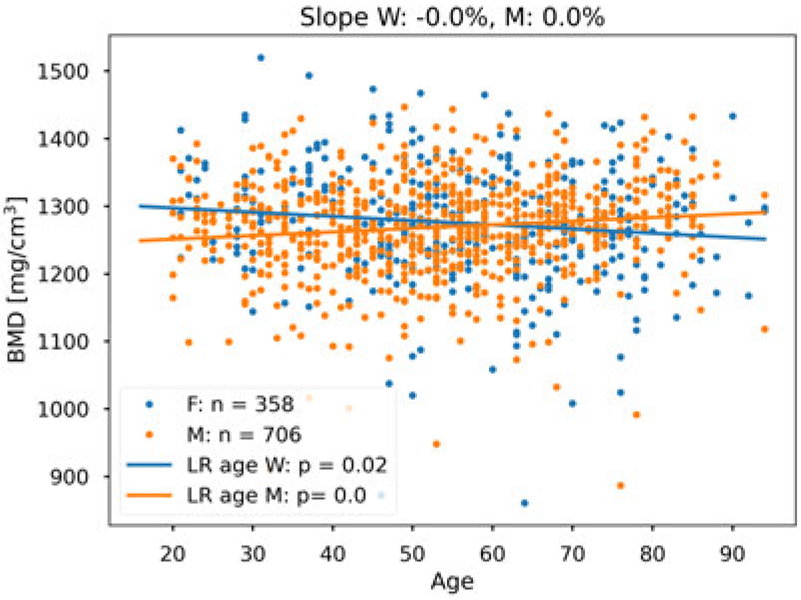

Figure 12: Linear regression between BMD and age for a point in the shaft cortex.

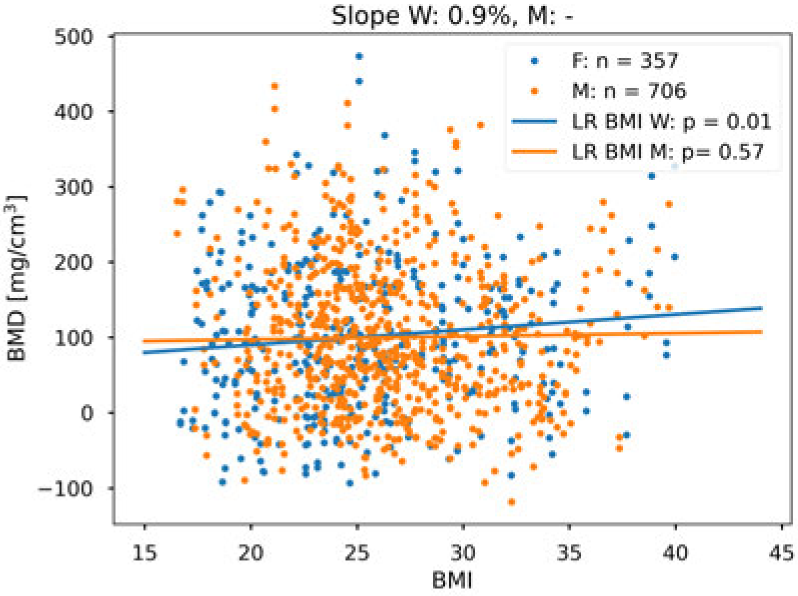

Figure 13: Linear regression between BMD and BMI for a point in the femoral neck.

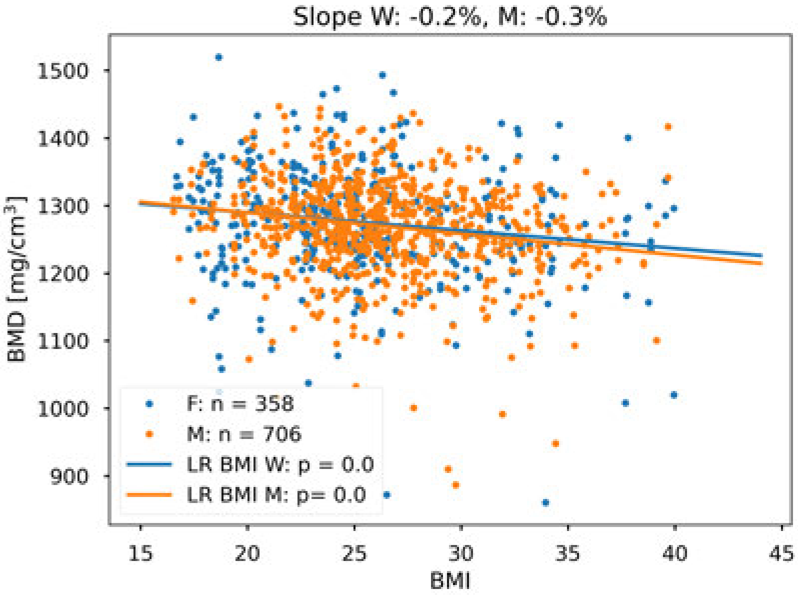

Figure 14: Linear regression between BMD and BMI for a point in the shaft cortex.
